# Tai Chi Chuan Exercise for Patients with Breast Cancer: A Systematic Review and Meta-Analysis

**DOI:** 10.1155/2015/535237

**Published:** 2015-02-22

**Authors:** Yuanqing Pan, Kehu Yang, Xiue Shi, Haiqian Liang, Fengwa Zhang, Qingfang Lv

**Affiliations:** ^1^Institute of Medical Psychology, Evidence-Based Medicine Center, Key Laboratory of Evidence Based Medicine and Knowledge Translation of Gansu Province, School of Basic Medical Sciences, Lanzhou University, Lanzhou 730000, China; ^2^Dean of Evidence-Based Medicine Center, Evidence-Based Medicine Center, Key Laboratory of Evidence Based Medicine and Knowledge Translation of Gansu Province, School of Basic Medical Sciences, Lanzhou University, Lanzhou 730000, China; ^3^Department of Internal Medicine, Gansu Rehabilitaition Center Hospital, Lanzhou, Gansu 730000, China; ^4^Department of Neurosurgery, The Affiliated Hospital of Logistics University of CAPF, Tianjin 300162, China; ^5^The Second Department of Gynaecology, Gansu Province People's Hospital, Lanzhou, Gansu 730000, China; ^6^Department of Radiology, The Tumor Hospital of Gansu Province, Lanzhou, Gansu 730050, China

## Abstract

*Objective*. Tai Chi Chuan (TCC) is a form of aerobic exercise that may be an effective therapy for improving psychosomatic capacity among breast cancer survivors. This meta-analysis analyzed the available randomized controlled trials (RCTs) on the effects of TCC in relieving treatment-related side effects and quality of life in women with breast cancer. *Methods*. RCTs were searched in PubMed, Embase, Web of Science, and Cochrane Library through April 2014. Data were analyzed on pathology (pain, interleukin-6, and insulin-like growth factor 1), physical capacity (handgrip, limb physical fitness, and BMI), and well-being (physical, social, emotional, and general quality of life). *Results*. Nine RCTs, including a total of 322 breast cancer patients, were examined. Compared with control therapies, the pooled results suggested that TCC showed significant effects in improving handgrip dynamometer strength, limb elbow flexion (elbow extension, abduction, and horizontal adduction). No significant differences were observed in pain, interleukin-6, insulin-like growth factor, BMI, physical well-being, social or emotional well-being, or general health-related quality of life. *Conclusion*. The short-term effects of TCC may have potential benefits in upper limb functional mobility in patients with breast cancer. Additional randomized controlled trials with longer follow-up are needed to provide more reliable evidence.

## 1. Introduction

Tai Chi Chuan (TCC) is a complementary and alternative therapy that has become a widespread exercise worldwide. Complementary and alternative medicines (CAM) are increasingly popular among female patients with breast cancer, and many use different forms of CAM to help manage their disease [1]. In 2002 alone, 62% of Americans used some form of CAM in the past 12 months, and approximately 60% of the urban female population over the age of 50 adopted TCC [2].

Several recent guidelines have made strong recommendations that some form of CAM should be adopted, even if those by the National Institute for Health and Clinical Excellence (NICE) tend to be cautious and stress the lack of evidence regarding CAM [3]. Considering 65 clinical guidelines, 48 make no mention of CAM, such as TCC. Notwithstanding, CAM has been extensively used in the treatment of breast cancer, even if evidence-based recommendations are lacking [3].

A meta-analysis assessing changes in pathological parameters following TCC in breast cancer patients has not been carried out thus far. While it has been reported that TCC leads to benefits in the reduction of both body mass index (BMI) [3] and pain [4, 5], other randomized trials found that TCC had no effects on the levels of IL-6, interferon-*γ* (IFN-*γ*), or insulin-like growth factor (IGF-1) [6, 7]. Additional studies have shown that following surgery for breast cancer TCC is associated with improved physical functioning and in particular of the shoulder joint (shoulder flexion and extension, exhibition, and spin) and muscle strength [8–10].

However, compared with conventional rehabilitation, psychological support therapy, and standard care in patients with breast cancer, whether or not TCC provides significant improvement in the quality of life remains an open question [11–13]. In some studies, TCC has been shown to reduce impairments related to breast cancer in parameters related to pathology physical activity and overall well-being, although there are conflicting reports in this regard.

To assess the efficacy of TCC in reducing breast cancer-related impairments, this comprehensive meta-analysis evaluated measures of pathology, physical activity, and overall well-being from the available randomized controlled trials (RCTs). This piece of information is useful to better understanding of the potential benefits of TCC in reducing treatment-related side effects and improving personal well-being in patients with breast cancer.

## 2. Materials and Methods

### 2.1. Search Strategy and Criteria of Selecting Studies

The PubMed (1966 to Nov., 2014), Embase (1974 to Nov., 2014), Cochrane Library (issue 11, 2014), and Web of Science (1974 to Nov., 2014) databases were queried using the search string (“breast neoplasms” (MeSH Terms) OR “breast neoplasm” (Title/Abstract) OR “breast cancer” (Title/Abstract) OR “breast tumor” (Title/Abstract) OR “breast neoplasms” (Title/Abstract) OR “breast cancers” (Title/Abstract) OR “breast tumors” (Title/Abstract)) AND (“TCC” (MeSH Terms) OR “TCC” (Title/Abstract)) AND (“Tai-ji” (MeSH Terms) OR “Tai-ji” (Title/Abstract)) AND (“Tai Chi” (MeSH Terms) OR “Tai Chi” (Title/Abstract)) AND (“Tai Ji Quan” (MeSH Terms) OR “Tai Ji Quan” (Title/Abstract)) AND (“Taiji” (MeSH Terms) OR “Taiji” (Title/Abstract)) AND (“Taijiquan” (MeSH Terms) OR “Taijiquan” (Title/Abstract)) AND (random^*^ OR “Clinical Trials as Topic” (Mesh) OR “Clinical Trial” (Publication Type)).

Female participants were eligible for inclusion if they were 1 aged 18 years or older; 2 had a history of breast cancer; and 3 received active breast cancer treatment. RCTs were included if they examined the effects of TCC on psychological symptoms (stress, anxiety, and/or depression), treatment-related symptoms (e.g., pain and/or fatigue symptoms), or regulation of inflammatory responses and other biomarkers (e.g., interleukin-6 (IL-6) and insulin-like growth factor 1 (IGF-1)). RCTs with different types of control groups were included.

### 2.2. Quality Assessment and Data Extraction

The methodological quality of studies was assessed independently by two reviewers (Yuanqing Pan and Fengwa Zhang) according to the criteria stated in the* Cochrane Collaboration Handbook* [17], which assess the quality of RCTs according to sequence generations, concealment of allocation, blinding of study participants and investigators, incomplete outcome data addressed, selective outcome reporting, and other sources of bias. Two reviewers (Haiqian Liang and Qingfang Lv) independently selected the RCTs based on the following characteristics: general information (first author, year of publication, and geographical location), study characteristics (sample size, mean age, and cancer stage), interventions (style, dosage, duration, and control group), and main outcome measures assessing physical deconditioning and inflammatory responses or biomarkers and treatment-related psychosomatic symptoms (pain and/or mood disturbances). Disagreements were rechecked by discussion with a third reviewer (Kehu Yang).

### 2.3. Statistical Analysis

Meta-analysis was carried out using Stata software (version 10.0; Stata Corp., College Station, TX, USA) [18]. For continuous outcomes, the standardized mean difference (SMD) and 95% confidence intervals (CI) were calculated. A fixed effects model was used if no statistically significant heterogeneity was identified, and a random effect model was adopted in the event of significant heterogeneity. Statistical heterogeneity between studies was evaluated using the chi-square test and *I* ^2^ statistics. Results were expressed as relative risk or mean difference with 95% CIs. A *P* < 0.05 was considered statistically significant. A metaregression approach was adopted to explore possible sources of heterogeneity among the RCTs; heterogeneities were estimated using Cochran's *Q*-test, with a *P* < 0.05 indicating statistically significant heterogeneity.

## 3. Results

### 3.1. Description of Studies

**Figure 1:**
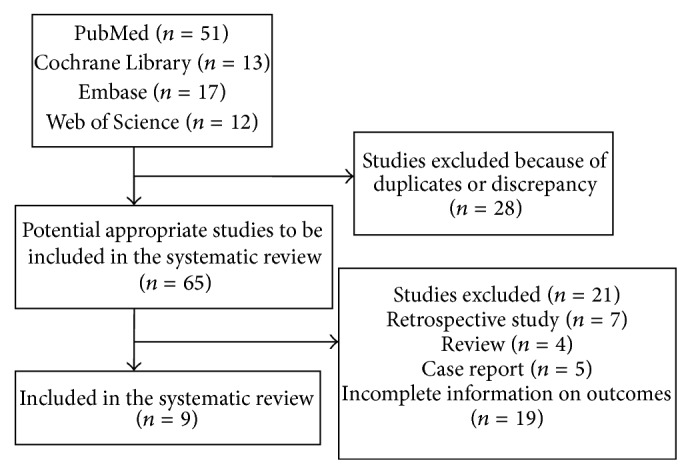
Flowchart of the results of the literature search.

A total of 93 studies were identified by searching databases. Of these, 28 were duplicates, and 57 did not meet the inclusion criteria. The baseline values in each trial were comparable, and nine RCTs were included in the meta-analysis [4, 6–10, 14–16] (Figure 1).

### 3.2. Study Characteristics

All nine RCTs were performed in USA [4, 6–10, 14–16]. The age of participants ranged from 49 to 65 years. One RCT [16] included patients with stage I-II breast cancer, one [8] included patients with stage 0–III tumors, and seven RCTs [4, 6, 7, 9, 10, 14, 15] included patients with stage I–III neoplasms. Participants in two RCTs [4, 7] underwent surgery and/or adjuvant radiation or chemotherapy during TCC intervention, in three RCTs [8–10, 14] patients underwent surgery, adjuvant radiation, chemotherapy, and hormone replacement therapy, in one RCT [15] patients received surgery and hormone therapy, and in two RCTs [6, 16] patients were receiving chemotherapy. The frequency of TCC varied from 2 to 3 times per week for 60–90 sessions, while the duration of treatment lasted from 10 weeks to 6 months. For four RCTs, participants in the control group were also provided with psychosocial therapy intervention [7–10]. The control groups in two RCTs received standard support therapy [4, 15]. In the remaining RCTs, the control groups received either health education [14], spiritual growth guidance, and standard health care [15] or usual care [6] (Table 1). Quality of life was measured using the Functional Assessment of Cancer Therapy-Breast (FACT-B) (version 4) [6, 16] and Functional Assessment of Chronic Illness Therapy-Fatigue survey (FACT) [8, 10]. Health-related quality of life 36 (HRQOL) [4] and medical outcomes study-short form (MOS SF-36) [14] self-report instruments were used to assess general health perceptions. Construct validity and internal consistency reliability (overall qualitative concerns in well-being of physical function social/family and emotion) were confirmed with satisfactory results for interscale correlations and known-group comparisons. A higher total score consistently indicated a more favorable health-related quality of life.

### 3.3. Methodological Quality of Studies

All nine RCTs [4, 6–10, 14–16] described the methods of adequate random sequence generation. Eight RCTs mentioned allocation concealment, and the blocks of one RCT [16] were concealed and sequences were stored in sealed, opaque, numbered envelopes. One RCT [14] adopted patient blinding of statisticians, one RCT [7] adopted patient blinding of therapists, and one RCT [6] reported blinding of assessors (Table 1).

The remaining RCTs did not mention whether selective outcomes reporting or other sources of bias were reported. Generally, in items of assessing the risk of bias with the Cochrane Book Scale, the methodological quality of trials was generally higher in those involving measures of similarity between groups at baseline, less than 15% dropouts, between-group statistical comparisons, outcome measures, and variability data (Table 2).

### 3.4. Outcomes considering Pathological Parameters

#### 3.4.1. Pain

**Figure 2:**
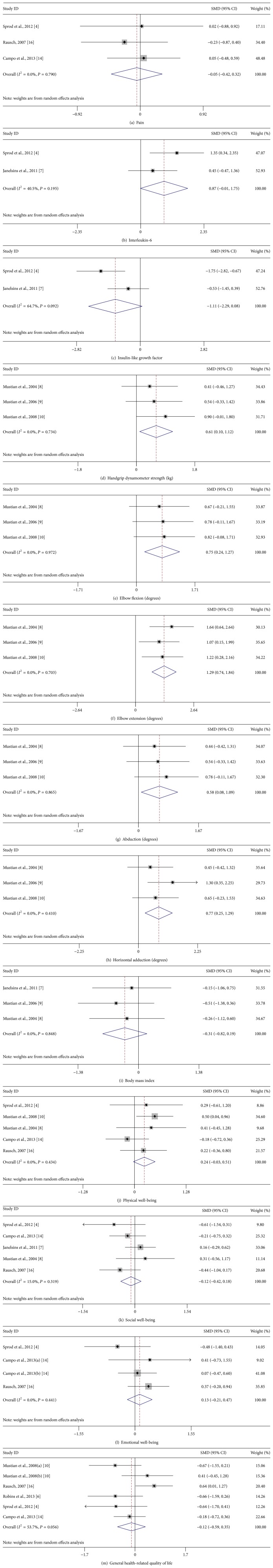
Forest plots of ORs with 95% CI for Tai Chi Chuan versus controls on (a) pain, (b) interleukin-6, (c) insulin-like growth factor, (d) handgrip dynamometer strength (kg), (e) elbow flexion (degrees), (f) elbow, extension (degrees), (g) abduction (degrees), (h) horizontal adduction (degrees), (i) body mass, (j) physical well-being, (k) social well-being, (l) emotional well-being, and (m) general health-related quality of life (random effect model). The width of the horizontal line represents the 95% CI of the individual studies, and the square proportional represents the weight of each study. The diamond represents the pooled OR and 95% CI.

Three RCTs [4, 14, 16] assessed changes in pain in the TCC intervention and control groups. The outcome measures used to assess pain were a health-related quality of life questionnaire [14, 16] and the SF-36 health survey [14]. No substantial heterogeneity was present between studies (*P* = 0.91; *I* ^2^ = 0%]. The pooled results suggested that the TCC group failed to improve pain compared with the control group (SMD −0.11; 95% CI −0.41, 0.18; *P* = 0.78] (Figure 2(a)) (Table 3).

#### 3.4.2. Interleukin-6

Two RCTs [4, 7] assessed the effects of TCC intervention on IL-6. There was no substantial heterogeneity between studies (*P* = 0.19; *I* ^2^ = 40.5%). Meta-analysis revealed that no differences were seen in IL-6 levels between the interventional and control groups (SMD 0.87; 95% CI −0.00, 1.75; *P* = 0.05) (Figure 2(b)) (Table 3).

#### 3.4.3. Insulin-Like Growth Factor 1

Two RCTs [4, 7] assessed the effect of TCC intervention on IGF-1 levels. Substantial heterogeneity was present between studies (*P* = 0.09; *I* ^2^ = 64.7%). Meta-analysis revealed that there were no differences between TCC intervention and the control group (SMD −1.10; 95% CI −2.29, 0.08; *P* = 0.06) (Figure 2(c)) (Table 3).

### 3.5. Outcomes Measuring Differences in Physical Parameters

#### 3.5.1. Handgrip Dynamometer Strength (kg)

Three RCTs [8–10] assessed the effects of TCC intervention on handgrip dynamometer strength. No substantial heterogeneity was present between studies (*P* = 0.73; *I* ^2^ = 0%). The pooled results suggested that the TCC group was associated with significant positive improvement in handgrip dynamometer strength compared with the control group (SMD 0.6; 95% CI 0.10, 1.11; *P* = 0.01) (Figure 2(d)) (Table 3).

#### 3.5.2. Limb Physical Fitness-Elbow Flexion (Degrees)

Three RCTs [8–10] assessed the effects of TCC intervention on elbow flexion of limb health-related physical fitness. No substantial heterogeneity was present between studies (*P* = 0.97; *I* ^2^ = 0%). The pooled results suggested that the TCC group had significantly greater improvement in elbow flexion compared with the control group (SMD 0.75; 95% CI 0.24, 1.26; *P* = 0.00) (Figure 2(e)) (Table 3).

#### 3.5.3. Limb Physical Fitness-Elbow Extension (Degrees)

Three RCTs [8–10] assessed effects of TCC intervention on elbow extension of limb health-related physical fitness. No substantial heterogeneity was present between studies (*P* = 0.07; *I* ^2^ = 7%). The pooled results suggested that the TCC group had significant improvement in elbow extension compared with the control group (SMD 1.29; 95% CI 0.74, 1.84; *P* = 0.00) (Figure 2(f)) (Table 3).

#### 3.5.4. Limb Physical Fitness-Abduction (Degrees)

Three RCTs [8–10] assessed the effects of TCC on abduction of limb health-related physical fitness. No substantial heterogeneity was present between studies (*P* = 0.86; *I* ^2^ = 0%). The pooled results suggested that the TCC group had significant improvement in limb abduction compared with the control group (SMD 0.58; 95% CI 0.07, 1.09; *P* = 0.02) (Figure 2(g)) (Table 3).

#### 3.5.5. Limb Health-Related Physical Fitness-Horizontal Adduction (Degrees)

Three RCTs [8–10] assessed the effects of TCC intervention on horizontal adduction of limb health-related physical fitness. No substantial heterogeneity was present between studies (*P* = 0.41; *I* ^2^ = 0%). The pooled results suggested that the TCC group was associated with significant improvement in limb abduction compared with the control group (SMD 0.77; 95% CI 0.25, 1.28; *P* = 0.00) (Figure 2(h)) (Table 3).

#### 3.5.6. Body Mass Index

BMI was assessed in three RCTs [7–9]. No substantial heterogeneity was present between studies (*P* = 0.84; *I* ^2^ = 0%). The pooled results suggested that the TCC group showed no difference in BMI compared with the control group (SMD 0.31; 95% CI −0.81, 0.19; *P* = 0.22) (Figure 2(i)) (Table 3).

### 3.6. Psychosomatic Well-Being

#### 3.6.1. Physical Well-Being

Physical well-being was assessed in five RCTs [4, 8, 10, 14, 16]. The outcome measures used to assess physical well-being included Functional Assessment of Chronic Illness Therapy-Fatigue survey [8, 10, 14], health-related quality of life 36 [4], and the SF-36 health survey [16]. No substantial heterogeneity was observed between studies (*P* = 0.43; *I* ^2^ = 0%). The pooled results suggested that there was no improvement in well-being in the TCC group compared with the control group (SMD 0.24; 95% CI −0.021, 0.51; *P* = 0.07) (Figure 2(j)) (Table 3).

#### 3.6.2. Social Well-Being

Social well-being was assessed in five RCTs [4, 7, 8, 14, 16]. The outcome measures used to assess physical well-being were the Rosenberg self-esteem scale [8], Functional Assessment of Chronic Illness Therapy-Fatigue survey [7], health-related quality of life 36 questionnaire [4], and FACT-B [14, 16]. Substantial heterogeneity was present between studies (*P* = 0.31; *I* ^2^ = 15%). The pooled results suggested that there was no improvement in social well-being in the TCC group compared with the control group (SMD −0.11; 95% CI −0.41, 0.18; *P* = 0.44) (Figure 2(k)) (Table 3).

#### 3.6.3. Emotional Well-Being

Social well-being was assessed in three RCTs [4, 14, 16]. The outcome measures used to assess physical well-being were health-related quality of life 36 [4], Center for Epidemiologic Studies depression scale [14], SF-36 health survey [16], and profile of mood states questionnaire [14]. No substantial heterogeneity was present between studies (*P* = 0.44; *I* ^2^ = 0%). The pooled results suggested that the TCC group did not improve emotional well-being compared with the control group (SMD 0.12; 95% CI −0.21, 0.47; *P* = 0.46) (Figure 2(l)) (Table 3).

#### 3.6.4. General Health-Related Quality of Life

FACT-B [4, 6, 10, 14, 16] and the health-related quality of life 36 questionnaire [15] were used to assess health-related quality of life in four RCTs. Substantial heterogeneity was present between studies (*P* = 0.05; *I* ^2^ = 53.7%). The pooled results suggested that the there was no improvement in the general health-related quality of life in the TCC group compared with the control group (SMD −0.12; 95% CI −0.59, 0.35; *P* = 0.61) (Figure 2(m)) (Table 3).

### 3.7. Metaregression Analyses

Heterogeneity was present between studies assessing the general health-related quality of life (*I* ^2^ = 68.1%). *P* values for all 11 factors (sample size, cancer staging, prior treatments, ethnicity, BMI, inclusion criteria for the TCC intervention group, intervention mentors, duration of intervention, TCC intervention program, establishment of the control group, and assessment tools for the quality of life) were greater than 0.5. No sources of heterogeneity were found for factors within and between studies (Table 4).

## 4. Discussion

The pooled results showed that the aerobic capacity of TCC improved muscle strength and flexibility of the upper limbs, without a reduction in BMI. It also failed to improve personal well-being and health-related quality of life and had no effect on changes in the levels of proinflammatory responses and other biomarkers.

### 4.1. Physical Parameters

A previous systematic review provided evidence that TCC has beneficial effects on a range of parameters of psychological well-being including depression, anxiety, general stress management, and exercise self-efficacy [5, 19]. However, other meta-analyses did not provide convincing evidence that TCC is effective for supportive breast cancer care in terms of improving the quality of life and psychological and physical clinical endpoints [11, 12].

The results of the present meta-analysis do not concur with those from systematic reviews, and the pooled results demonstrated a potential benefit of TCC in physical functioning. TCC also contributed to improvements in knee flexion through weight shifting, extending the head and trunk, and training balance systems to promote better steadiness, muscle strength, and flexibility [20, 21].

TCC is a novel integrative aerobic capacity that is associated with excellent adherence and attendance and has the potential to meet the unique needs of breast cancer patients by providing improvements in functional well-being. The complex interplay modes may provide a possible rationale for benefits in soft and connective tissues. These movements may induce local biochemical changes that modulate blood circulation, improve muscle flexibility, intensify the movement of lymphatic system, and loosen adherent connective tissue, which may improve reuptake of local nociceptive and inflammatory mediators.

Regular use of TCC after surgical intervention for breast cancer can obviously reduce the incidence of upper limb disorders and greatly improve rehabilitation. It should be emphasized that the generally positive findings observed herein must be interpreted cautiously. This is because participants may have been particularly amenable to exercise, and the results may not be applicable to those who are less amenable to TCC. All of the studies except one lasted only 12 weeks, and regional activity of the upper limbs must be relied upon rather than a general kinesthetic sense and strength in order to maintain the overall benefits on strength and flexibility after intervention is stopped.

### 4.2. Pathological Outcomes

Control over pain is thought to utilize at least two pathways in humans: the norepinephrine-serotonin pathway and the opioidergic pathway, and estrogen may play a role in modulation of the latter [22]. Aromatase inhibitor-associated musculoskeletal symptoms occur in approximately half of women undergoing treatment and lead to treatment discontinuation in 20−30% of cases [23]. In premenopausal breast cancer patients, lower estrogen levels have been associated with increased pain as well as with impairment of descending pain inhibitory pathways, which may be a risk factor for developing chronic pain and symptoms such as pain and stiffness [24]. Participants with aromatase inhibitor treatment alone or those undergoing chemotherapy met the present inclusion criteria at the 3-month time point of therapy. Interestingly, Henry et al. reported that the average pain threshold was significantly lower at the 3-month time point in patients who discontinued aromatase inhibitor therapy within 6 months [25].

The pain trends may be of particular clinical relevance for this population considering that the mean age in the present analysis was 53 years and thus in the context of profound chronic stress and estrogen deprivation. This suggests that, at least in some breast cancer patients, there may be an association between increased sensitivity to pain and impaired pain modulation prior to initiation of treatment. This pooled analysis showed that the beneficial response to TCC in secondary pain cannot be predicted by less efficient baseline pain modulation.

Ibrahim and Al-Homaidh reported that mortality was significantly reduced by TCC but only in breast cancer survivors with BMI <25 kg/m^2^ [26]. This may be related to the fact that some treatment-related side effects symptoms are related to excess body weight, especially in those patients undergoing hormone therapy (e.g., tamoxifen) or treatment-induced menopause [27]. These pooled results provide preliminary evidence that while TCC may not reduce BMI, the average height and weight for participants were not provided in baseline analyses [7, 8] and thus any effects on BMI are difficult to interpret. More specifically, the duration of present RCTs (12 weeks) and their moderate intense (2-3 times 60–90 minutes) may have been insufficient to detect significantly altered body composition.

The present results showed that there were no significant changes in the levels of IL-6, in agreement with previous studies [28]. Markers of inflammation cytokines, including IL-6 and IL-8, are expressed at high levels in the tumor microenvironment as well as during and after radiation and chemotherapy for breast cancer [29, 30]. IL-6 is rapidly produced by contracting skeletal muscle fibers and plays an important role in expression of anti-inflammatory myokines by inhibiting proinflammatory cytokine expression during regular exercise [31, 32]. Two studies have reported that 10–12 months of exercise interventions leads to lower circulating levels of IL-6; indeed, persistent inflammatory signaling can induce localized remodeling of tissue and production of insulin-signaling molecules, which can produce long-term changes in functional capacity (e.g., pain, muscle wasting, and weight gain) [33]. Irwin and Olmstead reported that the benefits of TCC had no impact on inflammatory markers until subjects fully learned all of the various movements by week 16 and were able to practice and consolidate skill acquisition by week 25 [34].

Hence, a longer-term intervention with TCC is necessary to observe some of its potential benefits. Given the effect sizes for this inflammation marker, the results should be interpreted with caution as effect sizes of IL-6 did not have sufficient statistical power to evaluate the effects of either TCC or IL-6 levels in the total sample also considering that there were heterogeneous levels of IL-6 at enrolment. IL-6 signaling pathways occur early in the inflammatory cascade with subsequent downstream effects on insulin-signaling molecules and insulin pathways (e.g., IGF-1), which may be related to weight gain, recurrence, and survival of patient with breast cancer.

Changes in other markers of inflammation might have been identified if the treatment and/or follow-up period was of longer duration by allowing more time to observe a decrease in this early signaling to drive subsequent activation of other insulin-signaling molecules.

TCC was not associated with any changes in the serum levels of IGF in the present meta-analysis, and it should be considered that the precise biological effects of IGF also depend on the cell growth state as well as other hormones and growth factors. Patients in this meta-analysis had undergone surgery, adjuvant radiation, chemotherapy, and hormonal therapy. This state of stress combined with the small sample size thus limits the ability to interpret these results in a biological context. Moreover, different control groups were used and the study design was also different in two RCTs (psychosocial therapy and standard support therapy), which may have masked the effects of TCC intervention [6, 15].

### 4.3. Well-Being

No significant improvements were seen in the quality of life dimensions assessed, such as general health perception, social functioning, and mental health or psychological well-being. These results are consistent with those of a meta-analysis conducted by Yan and Lee [11–13], which indicated that TCC has no impact on the mental health of cancer patients. Furthermore, Lee et al. showed that TCC failed to improve other quality of life subscales, except emotional well-being, and had no impact on other important clinical outcomes such BMI, bone mineral density, and muscle strength in patients with breast cancer following surgery [13].

A major challenge in integrating the results from prior RCTs is the heterogeneity in terms of outcome assessment of each study. Although the standardized pooled analysis was as consistent as possible, the potential for bias cannot be completely eliminated. Despite their shortcomings, assessments of subjective patient-reported outcomes such as health-related quality of life are still a key component of many clinical and research evaluations, and such differences can be considered to be clinically meaningful.

The present meta-analysis demonstrated that even if there is a lack of evidence of TCC in decreasing negative mood, enhancing self-esteem, and improving physical symptoms in patients with breast cancer, some trends were apparent through analysis of subscale scores. Moreover, physical function of patients was in decline with low SF-36 subscale scores at baseline [14] possibly limiting the power of these analyses.

### 4.4. External and Internal Validity

The studies included in this meta-analysis were conducted alternative to or complementary to methods used to cope with treatment-related side effects and improve quality of life in the US and involved patients with stages 0 to IIIb breast cancer and undergoing conventional treatment for breast cancer. Most studies have included predominantly well-educated women with relatively high socioeconomic status and good access to health care. However, while East Asia is the source for much interest in TCC, original published RCTs in this geographic region are very limited. The results of this meta-analysis are therefore not applicable to the vast majority of breast cancer patients and survivors in ethnically diverse samples from an underserved urban community.

There was a large risk of bias considering the studies included herein. It is, however, difficult to conduct blinding for participants or care providers in TCC studies, and blinding of outcome assessments is even more important. In this regard, only three studies reported on blinding of outcome assessors [6, 7, 14]. Randomization and/or allocation concealment were inadequately mentioned in the RCTs included, and the effects on health-related quality of life are not distinguishable from selection bias. Unfortunately, there were fewer than five studies in the previously published RCTs and systematic reviews evaluating the use of TCC as a complementary and alternative technique to improve the overall quality of life in patients with breast cancer. Because fewer than 10 studies were included herein, the effect of the meta-regression analysis used to assess publication bias is very limited. Furthermore, the risk of publication bias in the systematic review of RCTs based on small samples may be greater. Independently of whether the difference between the TCC intervention and control groups showed a statistically significant improvement in the quality of life of breast cancer patients following surgery, these RCTs may not provide an adequately precise estimate of efficacy.

### 4.5. Limitations

It is still difficult to draw firm conclusions since the studies reviewed herein are flawed in number of areas. First, the quality of the studies determines the quality of such analyses, and the studies included in this investigation had shortcomings in methodology. Significant heterogeneity in the RCTs was also present, such as expertise of practitioners, addressing the pluralism of TCC, description of the inconsistent TCC exercise sessions (frequency, intensity, and duration), primary outcome measures, and use of heterogeneous comparison groups.

Second, the present meta-analysis was based on nine published RCTs. The relatively small size of the study was a limitation since it restricts statistical power and may explain why some of the changes did not reach statistical significance. Furthermore, almost none of the RCTs in this meta-analysis measured follow-up beyond 12 weeks. Due to this, statistical power of this analysis cannot define the long-term efficacy of TCC.

Finally, if the blinding method of the nine RCTs had not been well implemented, a higher performance bias might have resulted. The RCTs reported that the reason for using the open-practice design in patients receiving a short-acting aerobic exercise may influence the results.

## 5. Conclusions

The current evidence demonstrates that in the short term TCC may have positive but moderate benefits in upper limb functional mobility in patients with breast cancer. There is no evidence to suggest that TCC is effective in improving psychosomatic status related to treatment-related symptoms. Because of the small number of studies and their methodological drawbacks, further large randomized controlled studies with longer follow-up period are needed to provide more reliable evidence.

## Figures and Tables

**Table 1:** Characteristics of the included studies.

Authors/year/country	Number of patients (TCC)	Number of patients (control)	Mean age of TCC group	Mean age of Control group	Status of cancer	Current treatment	Duration	Treatment group: intervention	Control group	Outcome measures/results
Campo et al., 2013, USA [14]	29	25	65.64 ± 7.15	66.54 ± 7.15	I–III	Treatment completed over 3 months before enrollment; undergone surgery, radiation, chemotherapy, and hormonal therapy	3 times 60 min, weekly for 12 weeks	TCC consisted of 19 specific simple, repetitive, nonstrenuous movements and 1 standing pose	60 min health education sessions occurred 3 times a week, over 12 weeks	Significant improvements in mental and physical benefits (SF-36) (*P* = 0.01) and CESD (*P* < 0.05)

Janelsins et al., 2011, USA [7]	9	10	54.33 ± 10.64	52.7 ± 6.67	I–IIIb	Treatment completed more than 1 month previously but less than 30 months before enrollment; undergone surgery, adjuvant radiation, and chemotherapy	3 times 60 min, weekly for 12 weeks	10 min warm-up stretching and basic Chi Kung; 15-move short-form sequence of Yang-style TCC	Psychosocial therapy: open-ended format that placed strong emphasis on teaching behavioral coping strategies, peer support, and group cohesion	Significant improvement in quality of life (FACIT) (*P* = 0.02) but not in the control group (*P* > 0.05); no significant improvements in both decreased IL-6 and IGF-1 (*P* > 0.05)

Mustian et al., 2004, USA [8]	11	10	52 ± 9	52 ± 9	0–III	Treatment completed 36 months before enrollment; undergone surgical treatment, adjuvant radiation, hormonal therapy, and/or chemotherapy	3 times 60 min, weekly for 3 months	10 min warm-up stretching and basic Chi Kung; 15-move short-form sequence of Yang-style TCC	Psychosocial therapy: open-ended format that placed strong emphasis on teaching behavioral coping strategies, peer support, and group cohesion (60 min, three times weekly for 12 weeks)	Significant improvements in both quality of life (FACIT-F) (*P* = 0.00) and self-esteem (RSE) (*P* < 0.01); significant improvements in handgrip dynamometer strength (kg) (*P* < 0.05), elbow flexion (degrees) (*P* < 0.05), elbow extension (degrees) (*P* < 0.05), abduction (degrees) (*P* < 0.05), and horizontal adduction (degrees) (*P* < 0.05)

Mustian et al., 2006, USA [9]	11	10	52 ± 9	52 ± 9	I–IIIb	Treatment completed more than 1 month but less than 30 months before enrollment; undergone surgery, adjuvant radiation, hormonal therapy, and/or chemotherapy	3 times/weekly for 12 weeks	10 min warm-up stretching and basic Chi Kung; 15-move short-form sequence of Yang-style TCC	Psychosocial therapy: open-ended format that placed strong emphasis on teaching behavioral coping strategies, peer support, and group cohesion (60 min, three times weekly for 12 weeks)	No significant improvements in BMI (*P* > 0.05); significant improvements in handgrip dynamometer strength (kg) (*P* < 0.05), elbow flexion (degrees) (*P* < 0.05), elbow extension (degrees) (*P* < 0.05), abduction (degrees) (*P* < 0.05), and horizontal adduction (degrees) (*P* < 0.05)

Mustian et al., 2008, USA [10]	11	10	52 ± 9	Unclear	I–IIIb	Treatment completed more than 1 month but less than 30 months before enrollment; undergone surgery, adjuvant radiation, hormonal therapy, and chemotherapy	3 times 60 min, weekly for 12 weeks	10 min warm-up stretching and basic Chi Kung; 15-move short-form sequence of Yang-style TCC	Psychosocial therapy: open-ended format that placed strong emphasis on teaching behavioral coping strategies, peer support, and group cohesion (60 min, three times weekly for 12 weeks)	Significant improvement in quality of life (FACIT) (*P* = 0.03) but not in the control group (*P* > 0.05); significant improvements in handgrip dynamometer strength (kg) (*P* < 0.05), elbow flexion (degrees) (*P* < 0.05), elbow extension (degrees) (*P* < 0.05), abduction (degrees) (*P* < 0.05), and horizontal adduction (degrees) (*P* < 0.05)

Peppone et al., 2010, USA [15]	7	9	53.8	52.9	I–IIIb	Treatment completed more than 1 month previously but less than 30 months before enrollment; undergone surgery, hormonal therapy	2 times/day; 20–30 min/session for 12 weeks	10 min warm-up stretching and basic Chi Kung; 15-move short-form sequence of Yang-style TCC	Standard support therapy	No significant improvement in bone metabolism (*P* = 0.17)

Rausch, 2007, USA [16]	15	14	49 (33–69)	49 (33–69)	I-II	Treatment completed more than 1 month previously but less than 30 months before enrollment; undergone chemotherapy	60 min at first, 90 min from second session, one time weekly for 10 weeks	Short form of TCC involving eight movements from experienced instructor	Spiritual growth and standard health care	Significant improvement in depression symptom (CESD) (POMS) (*P* < 0.05) and perceived stress (IES); significant improvement in quality of life (FACT-B) (*P* < 0.01)

Robins et al., 2013, USA [6]	37	36	50	50	I–IIIa	Receiving chemotherapy before and after enrollment	90 min each week for a total of 6 months	8 TCC movements focused on repetitive, attention, flexibility, and mind-body relaxation	Usual care	Significant improvement in perceived stress scores (IES) (*P* < 0.0001) and quality of life (FACT-B) (*P* < 0.0001) in the intervention group

Sprod et al., 2012, USA [4]	9	10	54.33 ± 3.55	52.7 ± 2.11	I–IIIb	Treatment completed more than 1 month but less than 30 months before enrollment; undergone surgery, adjuvant radiation, and/or chemotherapy	3 times/week; 60 min/session 12 weeks	10 min warm-up, 40 min of Yang-style TCC using the 15-move short form which is traditional 15 moves and 104-move long form, and 10 min breathing, imagery, and meditation	Standard support therapy	Significant improvement in quality of life, physical functioning (HRQOL) (*P* = 0.030), physical role limitations (HRQOL) (*P* = 0.023), social functioning (HRQOL) (*P* = 0.020) (IL-6) (IGF-1)

Activities of daily living (ADL); body mass index (BMI); Center for Epidemiologic Studies depression scale (CESD); Functional Assessment of Cancer Therapy-Breast (FACT-B); Functional Assessment of Chronic Illness Therapy-Fatigue survey (FACIT); health-related quality of life 36 (HRQOL); Impact of Events Scale (IES); interleukin-6 (IL-6); insulin-like growth factor-1 (IGF-1); profile of mood states (POMS); Rosenberg self-esteem scale (RSE); World Health Organization quality of life questionnaire (WHOQOL-BREF); SF-36 health survey (SF-36); Tai Chi Chuan (TCC).

**Table 2:** Methodological quality of included studies.

Reference	Randomization	Allocation concealment	Blinding	Incomplete outcome data	Selective outcome reporting	Other Sources of bias
Campo et al., 2013, USA [14]	Randomized using random permuted blocks	Mention	Yes (statisticians)	Yes	Unclear	Unclear
Janelsins et al., 2011, USA [7]	Randomized via mailed letters	Mention	Yes (therapists)	Unclear	Unclear	Unclear
Mustian et al., 2004, USA [8]	Flipping of a coin	Mention	Mention	Unclear	Unclear	Unclear
Mustian et al., 2006, USA [9]	Flipping of a coin	Mention	Mention	No	Unclear	Unclear
Mustian et al., 2008, USA [10]	Flipping of a coin	Mention	Mention	Unclear	Unclear	Unclear
Peppone et al., 2010, USA [15]	Randomized using computer-generated random list	Mention	Mention	Unclear	Unclear	Unclear
Rausch, 2007, USA [16]	Randomized via mailed letters	Opaque, numbered envelopes	Mention	Unclear	Unclear	Unclear
Robins et al., 2013, USA [6]	Randomized using computer-generated random list	Mention	Yes (assessor)	Yes	Unclear	Unclear
Sprod et al., 2012, USA [4]	Flipping of a coin	Mention	Mention	No	Unclear	Unclear

**Table 3:** Effect sizes of Tai Chi Chuan versus control interventions.

Outcome	Number of studies	Number of patients	Standardized mean difference [95% confidence interval]	Heterogeneity *P* value	*I* ^2^	Test for overall effect *P* value
Pain	2 [4, 14, 16]	112	−0.11 (−0.41, 0.18 )	*P* = 0.79	0.00%	*P* = 0.78
Interleukin-6	2 [4, 7]	35	0.87 (−0.00, 1.75)	*P* = 0.19	40.5%	*P* = 0.05
Insulin-like growth factor	2 [4, 7]	35	−1.10 (−2.29, 0.08)	*P* = 0.09	64.7%	*P* = 0.06
Handgrip dynamometer strength (kg)	3 [8–10]	63	0.60 (0.10, 1.11)	*P* = 0.73	0.00%	*P* = 0.01
Elbow flexion (degrees)	3 [8–10]	63	0.75 (0.24, 1.26)	*P* = 0.97	0.00%	*P* = 0.00
Elbow extension (degrees)	3 [8–10]	63	1.29 (0.74, 1.84)	*P* = 0.07	0.00%	*P* = 0.00
Abduction (degrees)	3 [8–10]	63	0.58 (0.07, 1.09)	*P* = 0.86	0.00%	*P* = 0.02
Horizontal adduction (degrees)	3 [8–10]	63	0.77 (0.25, 1.28)	*P* = 0.41	0.00%	*P* = 0.00
Body mass index	3 [7–9]	61	0.31 (−0.81, 0.19)	*P* = 0.84	0.00%	*P* = 0.22
Physical well-being	4 [4, 8, 10, 14, 16]	216	0.24 (−0.02, 0.51)	*P* = 0.43	0.00%	*P* = 0.07
Social well-being	4 [4, 7, 8, 14, 16]	213	−0.11 (−0.41, 0.18)	*P* = 0.31	15%	*P* = 0.44
Emotional well-being	4 [4, 14, 16]	137	0.12 (−0.21, 0.47)	*P* = 0.44	0.00%	*P* = 0.46
General health-related quality of life	4 [4, 6, 10, 14, 16]	178	−0.12 (−0.59, 0.35)	*P* = 0.05	53.7%	*P* = 0.61

**Table 4:** Effect sizes of metaregression analysis in overall quality of life.

	Constant	Coefficient	SE	*T*-value	*P* value	95% confidence interval	
						UL	LL
General health-related quality of life							
Age	−0.45	0.20	0.32	0.61	0.57	−0.71	1.11
Stage of cancer	−0.34	0.82	0.26	0.31	0.76	−0.65	0.81
Current treatment	−0.06	−0.05	0.15	−0.35	0.75	−0.48	0.38
Race	0.07	−0.06	0.20	−0.34	0.75	−0.620	0.48
BMI	0.75	−0.36	0.20	−1.78	0.19	−0.94	0.20
Tai Chi Chua inclusion criteria	0.16	−0.11	0.20	0.55	0.60	−0.67	0.45
Tai Chi Chua instructor	−0.14	0.00	0.28	0.02	0.99	−0.78	0.79
Duration	−0.12	−0.00	0.36	−0.02	0.98	−1.02	1.00
Tai Chi Chuan movements	0.16	−0.11	0.20	−0.55	0.61	−0.67	0.45
Control group	0.16	−0.11	0.20	−0.55	0.61	−0.67	0.45
Scale	−0.23	0.03	0.20	0.18	0.87	−0.51	0.59
